# *In vitro* effects of etanercept on the development of different T cell subsets in patients with juvenile idiopathic arthritis

**DOI:** 10.1186/1546-0096-9-S1-P112

**Published:** 2011-09-14

**Authors:** Rolando Cimaz, Lorenzo Cosmi, Laura Maggi, Gabriele Simonini, Teresa Giani, Ilaria Pagnini, Veronica Santarlasci, Manuela Capone, Valentina Querci, Francesco Liotta, Enrico Maggi, Francesco Annunziato

**Affiliations:** 1 AOU Meyer and Careggi, Firenze, Italy

## Background

Recently, we found that CD4+CD161+ T lymphocytes showing transient nature of the Th17 phenotype are present in the synovial fluid of patients with JIA, and that their accumulation positively correlates with parameters of inflammation, supporting the hypothesis that these cells may play a role in disease activity.

## Aim

In the present study, we assessed the ability of etanercept to influence the *in vitro* development of different T cell subsets in JIA patients. In particular, we have evaluated the ability of peripheral blood (PB) and synovial fluid (SF) lymphocytes from patients followed in our Unit, to proliferate and produce cytokines in response to polyclonal (antiCD3/antiCD28) stimulation in presence or in absence of etanercept.

## Methods

Lymphocytes from PB and SF have been cultured in the presence of etanercept (5 µg/ml), and proliferative responses were evaluated as thimidine uptake and % of bromodeoxyuridine positive cells. Cell phenotype by flow cytometry and cytokine production (% of producing cells) in CD4+ cells were also assessed.

## Results

We have initially studied 11 patients with oligoarticular or polyarticular JIA who underwent therapeutic arthrocentesis, with concomitant venipuncture for routine blood tests. Administration in culture of etanercept did not significantly affect the proliferative response to aCD3/aCD28 stimulation of both PB- and SF-derived total CD4+ lymphocytes, whereas the presence in culture of etanercept increased the frequency of proliferating CD4+CD161+ lymphocytes. Accordingly, etanercept *in vitro* administration increased the frequency of total IL-17-producing CD4+ T cells, leaving the IFN-gamma-producing ones unaffected.

## Conclusions

These *in vitro* preliminary results seem to indicate that etanercept is able to increase the frequency of IL-17 producing cells by favouring their proliferation.

